# Integrating Routine Hematological and Extended Inflammatory Parameters as a Novel Approach for Timely Diagnosis and Prognosis in Sepsis Management

**DOI:** 10.3390/diagnostics14090956

**Published:** 2024-05-02

**Authors:** Sianny Herawati, I Ketut Agus Somia, Sully Kosasih, I Nyoman Wande, Jethro Felim, I Made Dwi Payana

**Affiliations:** 1 Department of Clinical Pathology, Faculty of Medicine, Universitas Udayana, Bali 80114, Indonesia; wandenyoman@gmail.com; 2 Department of Internal Medicine, Faculty of Medicine, Universitas Udayana, Bali 80114, Indonesia; agus.somia@unud.ac.id; 3 PT Sysmex Indonesia, Jakarta 12950, Indonesia; kosasih.sully@sysmex.co.id; 4 Clinical Pathology Residency Education Program, Faculty of Medicine, Universitas Udayana, Bali 80114, Indonesia; jethrofelim@yahoo.com (J.F.); dwipayana17@gmail.com (I.M.D.P.)

**Keywords:** sepsis, extended inflammatory parameters, mortality

## Abstract

Sepsis is one of the major causes of morbidity and mortality in hospitals, especially in low- and middle-income countries, and represents a challenge to health care providers to carry out early detection, and accurate diagnosis and prognosis with cost-effective diagnostic tools. An observational prospective study was conducted from December 2021 to December 2022 to investigate the extended inflammatory parameters (EIPs) for sepsis management and analyze the survival of septic patients in the emergency unit, intensive care unit (ICU) and inpatient ward. Patients suspected of having sepsis underwent a sequential organ failure assessment (SOFA) evaluation and had blood drawn for complete blood counts (CBCs). Significant changes were observed in various CBC parameters and EIPs, and the sepsis group was followed up with for 30-day mortality. The study highlighted a significant difference yet strong discriminatory power to differentiate sepsis with an AUC of 0.924 against the non-sepsis group and an AUC of 0.991 against the healthy control group using combination of white blood cells and EIPs. Furthermore, the study showed good predictive ability for 30-day mortality with a hazard ratio of 2.311. In summary, this study provides evidence that the utilization of EIPs may be valuable in diagnosing and predicting patient outcomes, and thus will be beneficial for sepsis management in the hospital.

## 1. Introduction

Sepsis, a systemic inflammatory response triggered by infection, poses a significant global health challenge, with an estimated incidence of 48.9 million cases and a mortality rate of approximately 19.7%, responsible for more than 11 million sepsis-related deaths [1,2]. The condition may arise from various infections, including bacteria, fungi, viruses, and parasites, and if left untreated leads to organ failure and death. Several risk factors for the development of sepsis have also been identified, including male gender, race, age, medical comorbidities, alcohol abuse, lower socioeconomic status, and cold temperature factors. In Indonesia, the economic burden associated with sepsis has risen, particularly affecting patients with multifocal infections and single-focal lower respiratory tract infections [3].

In resource-limited settings such as Indonesia, the diagnosis of sepsis faces significant challenges, primarily due to the absence of well-established biomarkers. The lack of precise markers makes early detection and accurate diagnosis a formidable task, impacting the timely initiation of life-saving interventions [4]. In these settings, where access to advanced medical technologies is often constrained, healthcare providers grapple with the need for cost-effective and easily deployable diagnostic tools, in which sepsis, a time-sensitive condition, requires efficient and reliable markers for swift identification [5,6]. The scarcity of such markers not only hinders the prompt diagnosis but also contributes to the high mortality rates associated with sepsis. Addressing these challenges necessitates a concerted effort to develop accessible and affordable diagnostic solutions tailored to resource-limited environments, ensuring that even in regions with constrained resources, patients can benefit from timely and effective sepsis management.

The sepsis diagnosis criteria [7,8], established in 2016 by the European Society of Intensive Care Medicine and the Society of Critical Care Medicine, introduced the sequential organ failure assessment (SOFA) score system, emphasizing its broader role beyond the systemic inflammatory response syndrome (SIRS) criteria by incorporating response to infection and organ damage, classified as SOFA ≥ 2. To enhance clinical applicability, a “quick SOFA” or qSOFA assessment has been developed, indicating organ dysfunction with a qSOFA value ≥2 based on specific criteria such as respiratory rate, changes in consciousness, and systolic blood pressure [9,10,11].

The latest update of the Surviving Sepsis Campaign’s adult sepsis guidelines acknowledges sepsis as life-threatening organ dysfunction resulting from a dysregulated host response to infection, aligning with the Sepsis-3 consensus definition. Furthermore, it specifically addresses the importance of patient care post-discharge from the ICU. These updates demonstrate a broader representation of geographic and gender diversity compared to earlier guidelines [12,13].

The implementation of SOFA and qSOFA in Indonesia encounters several challenges. The intricacy arises from the diverse healthcare landscape, where limited resources, varying levels of medical expertise, and disparities in healthcare infrastructure hinder seamless integration [3]. The comprehensive nature of the SOFA score, encompassing multiple organ systems and laboratory parameters, demands a robust healthcare system that may be lacking in certain regions of Indonesia. Moreover, the qSOFA criteria, designed for rapid assessment, may face hurdles in consistency due to variations in healthcare practices across different provinces and settings.

The imperative for cost-effective biomarkers in the context of sepsis diagnosis cannot be overstated. Cost-effective biomarkers not only facilitate early and accurate identification of sepsis but also contribute significantly to reducing the economic burden on healthcare systems and patients [14,15]. Prioritizing the development and utilization of biomarkers that are both clinically effective and economically viable ensures that even resource-limited settings benefit from improved diagnostic capabilities, ultimately leading to better patient outcomes and more efficient allocation of healthcare resources [16,17].

Exploring the measurement of immune response as a feasible solution for early sepsis detection holds significant promise [18]. Leveraging routine hematology, which is a widely accessible and established diagnostic tool [19], can offer an efficacious approach in this endeavor [20]. Monitoring key indicators within the immune response through routine hematology parameters can provide valuable insights into the body’s reaction to infection, enabling timely identification of potential sepsis cases. This approach not only aligns with the need for cost-effective diagnostic solutions, crucial in resource-limited settings like Indonesia, but also underscores the practicality of utilizing existing infrastructure for more efficient and widespread sepsis screening. 

Previous research has highlighted the utility of various cell population data (CPD) in measuring immune response, particularly in cases with viral origins [21,22]. This has led to the development of an intensive care infection score (ICIS), mirroring the functionality of procalcitonin (PCT) in identifying bacterial infections in hospitalized patients [23,24]. Routine hematology tests offer notable advantages, including swift turnaround time and minimal sample requirements. The expeditious nature and minimal sample needs constitute key benefits of these tests. Integrating pertinent immune response parameters further enables real-time measurement in critical cases of inflammation and infection. This combined approach enhances the effectiveness of routine hematology tests, particularly in time-sensitive situations. 

The primary objective of this study is to assess diagnostic and prognostic potential of various routine and research parameters provided by the Sysmex XN 1000 hematology analyzer in cases of sepsis. As the initial diagnostic tool for patients presenting with febrile illnesses in emergency settings and a routine assessment for those in intensive care, studying hematology profiles across diverse patient groups aims to identify potential combination biomarkers indicative of hematological inflammation. This pursuit holds the potential to enhance the specificity and measurability of biomarkers, contributing to more accurate sepsis diagnosis and prognosis.

## 2. Materials and Methods

### 2.1. Study Design 

An observational prospective study was carried out at Prof. Dr. I.G.N.G Ngoerah Hospital, in the emergency unit, intensive care unit (ICU), high-dependency inpatient ward, and inpatient ward. The high-dependency inpatient ward includes a group of patients in the inpatient ward that require more intense monitoring. Approval for the study was granted by the Human Research Ethics Committee of the Faculty of Medicine, Universitas Udayana (No. 2848/UN14.2.2.VII.14/LT/2021), covering the period from December 2021 to December 2022. To address sample size sufficiency, a prevalence of 31.2% of septic events occurring within the ICU was taken into consideration [25]. This was determined to require a minimum sample size of 28 septic cases, factoring in 10% dropout. Patient selection for the study adhered to specific inclusion criteria, with individuals suspected of having sepsis being recruited into the sepsis group. Additional inclusion criteria stipulated that participants must be above 18 years of age, and qSOFA measurements for this group were confirmed to be greater than or equal to 2. 

For the non-sepsis group, patients were recruited from the high-dependency inpatient ward, inpatient ward, emergency care, and ICU. Subject selections were based on attending physicians’ perspectives of high suspicion of sepsis, and subsequently, the qSOFA was established. In all cases, the qSOFA was less than 2. These patients were continuously monitored throughout their stay and confirmed to remain non-septic. Exclusion criteria encompassed malignancy, HIV infection, pregnancy, and incomplete documentation. All patients included in the study provided written informed consent. Seven septic cases were excluded in deriving our model due to an incomplete dataset. The final analysis comprised 39 patients with sepsis and 39 patients without sepsis, after filtering out cases that did not meet the specified criteria. The stringent inclusion criteria were implemented to ensure the credibility of the study and the accuracy of the results obtained. In addition, a control group involving healthy subjects was included for comparison to establish reference intervals, thereby providing valuable context for clinical interpretations. Post analysis, a power calculation was performed on selected hematological parameters used in this study to ascertain the statistical significance. 

### 2.2. Sample Collection and Analysis

In this trial, residual samples were utilized, involving the use of 3 mL peripheral blood samples collected using Becton Dickinson (BD, Franklin Lakes, NJ, USA) vacutainer K2-EDTA. A complete blood count (CBC) was conducted using an XN-3000 hematology analyzer (Sysmex Corp., Kobe, Japan). All samples were processed within an hour of collection, including an additional RET channel on the hematology analyzer. For serial monitoring of the CBC, another 3 mL of whole blood was obtained on day 3. The extraction of cell population data (CPD), advanced clinical parameters, and extended inflammatory parameters (EIPs), namely, Neut-RI, Neut-GI, AS-Lymph, and RE-Lymph, was carried out manually. This involved exporting sample runs from the information processing unit (IPU) of the XN analyzer. 

The selected hematology parameters were computed and systematically compared across septic, non-septic, and healthy control populations. These parameters were combined to serve as an indirect metric for assessing immune response. To ascertain clear diagnostic accuracy, a multiple logistic regression analysis was employed. The establishment of this model aimed to prognosticate the likelihood of a septic episode in patients upon admission to the ICU. By leveraging these combined hematology parameters within a predictive framework, the model contributes to a more nuanced understanding of immune response dynamics, facilitating early identification and proactive management of sepsis in critical care settings.

### 2.3. Patient Follow-up for 30-Day Mortality 

In the context of post-sepsis research against the usefulness of predictive hematology parameters, a robust patient follow-up protocol was drafted for comprehensively evaluating the aftermath of septic episodes. The 30-day mortality follow-up entails a systematic tracking and evaluation of patients post-treatment, facilitating the monitoring of recovery, the identification of potential complications, and the measurement of the long-term impact of sepsis on patient outcomes. Employing the model derived from distinguishing septic and non-septic cases, we established robust predictive capabilities within this 30-day mortality period. The model’s predictive ability was determined by setting a cut-off at the 60th percentile of results obtained for septic cases in the prognostication model. To visually evaluate the effectiveness of this setup, a Kaplan–Meier (KM) plot was constructed, providing valuable insights into the predictive performance of the model over the specified follow-up period.

### 2.4. Statistical Analysis

The data obtained were reported as means with standard deviations (SDs) for numerical variables and frequencies (n) with percentages (%) for categorical variables, unless otherwise specified. Hematological parameters and EIP results with normality ascertained across different study groups were compared using the one-way analysis of variance (ANOVA); otherwise, the Kruskal–Wallis test was employed. Descriptive statistics and a multiple logistic regression model were computed using the Statistical Package for Social Sciences (SPSS) Statistics for Windows (Version 26.0, IBM Corp, Armonk, NY, USA). The repeated-measures ANOVA was applied to analyze the serial measurements of numeric variables of EIPs, including Neut-RI, Neut-GI, AS-Lymph, and RE-Lymph, within the sepsis cohort from day 0 to day 3. Receiver operating characteristic (ROC) curve analysis was conducted, and the optimal cutoff, sensitivity, and specificity were determined using SPSS. Survival probabilities for septic patients were estimated through the Kaplan–Meier method, and the hazard ratio was determined using Mantel–Haenszel analysis. Statistical significance was considered if the *p*-value was less than 0.05. 

## 3. Results

### 3.1. Subject Demographics and Extended Inflammatory Parameter Profiles 

**Figure 1:**
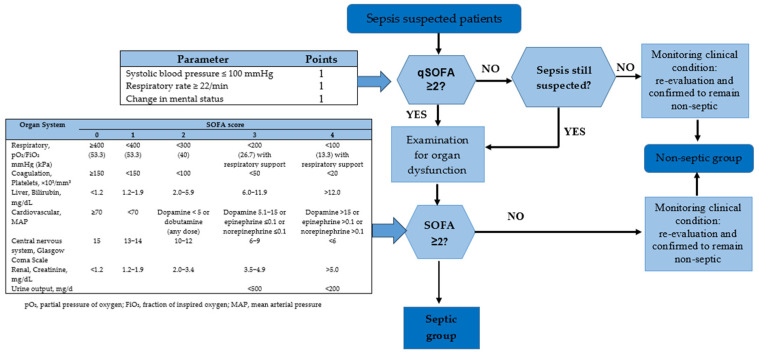
Patient recruitment workflow [2,7].

The study encompassed a total of 78 patients and 33 healthy specimens, with corresponding patient demographics detailed in Table 1. Patient classification into sepsis and non-sepsis groups was determined through clinical adjudication, as depicted in Figure 1. In the sepsis group, there were 20 (51.3%) male and 19 (48.7%) female patients, with a mean age of 60.8 years and a standard deviation (SD) of 14.8 years. The non-sepsis group comprised 24 (61.5%) male and 15 (38.5%) female patients, with a mean age of 53.0 years and an SD of 16.3. The healthy control group included 23 (69.7%) males and 10 (30.3%) females, with mean age of 33.7 years and SD of 6.1. Within the sepsis patients, only 5 (12.8%) yielded positive blood culture results, while 34 (87.2%) exhibited negative blood culture outcomes. In the sepsis group, we observed mostly respiratory infection cases and a few of gastrointestinal and urinary tract infection, with 5 positive cultures of *Pseudomonas putida*, *E. coli*, and *Streptococcus agalactiae*. Conversely, within the non-sepsis group, the conditions ranged from non-communicable diseases to trauma cases.

Beyond the routine CBC, additional parameters were incorporated, including four EIPs associated with the activation of lymphocytes and neutrophils. Additionally, parameters such as RET-He, Delta He, and RBC-He, reflecting the production and maturation of blood cells, were included in the analysis. The study comprehensively analyzed various hematological parameters, revealing that the sepsis group exhibited significantly higher levels of total white blood cell count (TWBC), neutrophil count (NEUT#), neutrophil percentage over TWBC (NEUT%), immature granulocyte count (IG#), neutrophil reactivity intensity (Neut-RI), neutrophil granularity intensity (Neut-GI), antibody synthesizing lymphocytes (AS-Lymph), and reactive lymphocytes (RE-LYMPH) compared to the non-sepsis groups, as illustrated in Table 1. Additionally, the monocyte percentage relative to TWBC (Mono%) exhibited statistically significant lower mean values in the septic group compared to the non-septic and healthy cohorts.

**Figure 2:**
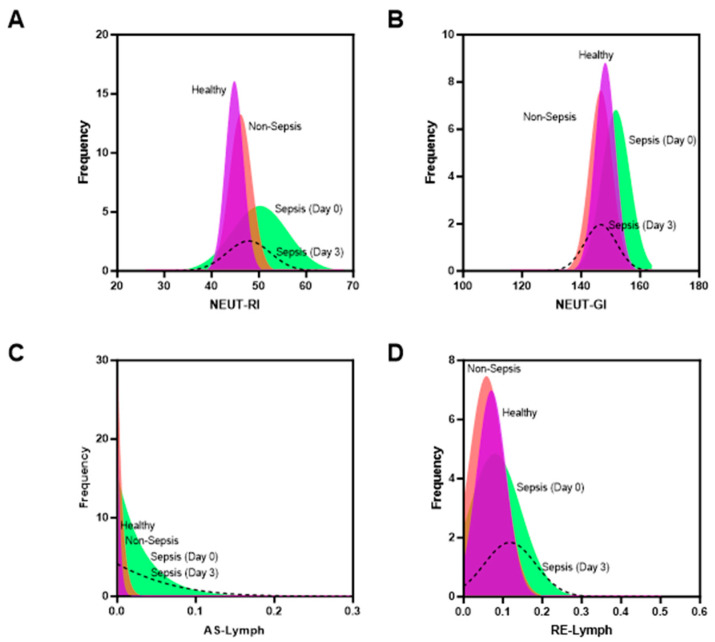
Distribution curve of hematology-extended inflammatory parameters in sepsis and non-sepsis groups. (**A**) Neut-RI; (**B**) Neut-GI; (**C**) AS-Lymph; (**D**) RE-Lymph.

Figure 2 illustrates the frequency distributions of EIPs associated with both septic and non-septic cases. The inclusion of a reference curve for healthy individuals facilitates the assessment of overlaps within diverse study populations. To elucidate the temporal evolution of infection, a restricted dataset featuring day 3 results for septic cases is presented. Instances where day 3 results were censored can be attributed to either patient mortality or unavailability of blood specimens.

Consistently across all cases, a significant rightward shift in EIP results for septic cases in comparison to non-septic cases is evident. The statistical significance of these disparities is detailed in Table 1. Of particular note are the heightened variances observed in Neut-RI (Figure 2A) and AS-Lymph (Figure 2C) for septic cases. To validate these findings, raw datasets are graphically represented in Supplementary Figure S1, emphasizing the intricate interplay of various white cell types in the context of sepsis.

Crucially, with the exception of Neut-GI, a diminution in the variance of EIPs is noted at day 3 of measurement. Intriguingly, mean levels of Neut-RI and Neut-GI (Supplementary Figure S1A,B) revert to levels more proximal to those of non-septic cases, while AS-Lymph and RE-Lymph remain elevated (Supplementary Figure S1C,D). This underscores the nuanced and dynamic nature of immune responses during the course of sepsis.

### 3.2. Discriminatory Power of Hematology Parameters to Identify Immune Responses between Groups

**Figure 3:**
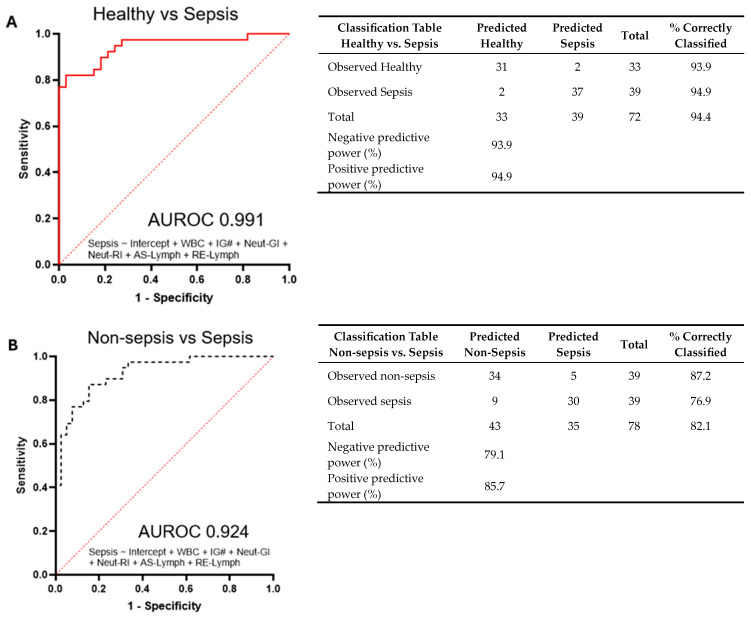
Receiver operator curves for combination of hematological parameters: Intercept + WBC + IG# + Neut_GI + Neut-RI + AS-Lymph + RE-Lymph for (**A**) healthy vs. sepsis and (**B**) non-sepsis vs. sepsis cases. The cutoff value is set at 0.5 and the tables summarize the accuracy of the curve plotted.

In the context of sepsis, the simultaneous assessment of neutrophils and lymphocytes enables a more nuanced understanding of the host’s immune status. A multiple logistic regression model was thus formulated, integrating parameters TWBC + IG# + EIP (Neut-GI + Neut-RI + AS-Lymph + RE-Lymph) and utilizing the default cutoff level at 0.5. This model demonstrated strong discriminatory prowess, effectively distinguishing septic cases from other cohorts, as illustrated in Figure 3. Elevated white cell counts, immature granulocytes indicative of infection and inflammation, and heightened activation levels in neutrophils and lymphocytes collectively presented compelling evidence of discriminatory capability on day 0 for ICU patients. The results, quantified through the area under the ROC (AUROC) curve, reached an impressive 0.991. This signifies the model’s exceptional ability to discern sepsis cases from healthy counterparts, achieving a noteworthy negative predictive power of 93.9% and positive predictive power of 94.9%. Additionally, the AUROC value for discriminating septic cases from non-septic cases was 0.924, with an accompanying negative predictive power of 79.1% and positive predictive power of 85.7%. These outcomes underscore the robust discriminatory potential of these hematological parameters, highlighting their clinical significance as complementary and cost-effective laboratory assays to support early sepsis detection.

### 3.3. Prognostic Ability of Combined Hematological Parameters towarods 30-Day Mortality

**Figure 4:**
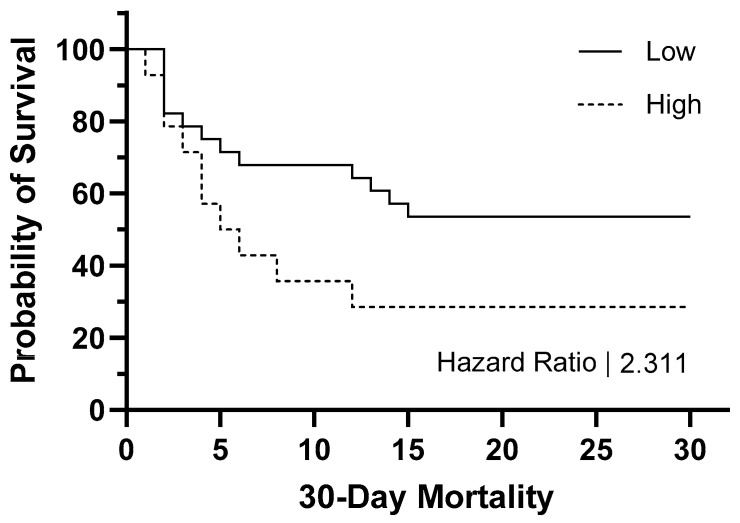
Kaplan–Meier predictive analysis within a 30-day monitoring period. Patients with high levels of combination of hematological parameters: WBC + IG# + Neut_GI + Neut-RI + AS-Lymph + RE-Lymph on day 0 show worse outcomes.

The evaluation of 30-day mortality serves as a crucial metric in the comprehensive management of sepsis. In line with this, our hypothesis posits that the integrated hematological parameter model can significantly contribute to the prognosis of such cases within the septic population. Patients were systematically followed up with as part of the hospital registry, enabling the identification of potential complications or persistent challenges in the post-septic phase. Implementing a 60–40 split within the septic study group, corresponding to a cutoff value of 0.95 derived from the model designed for distinguishing septic and non-septic cases, allowed for the stratification of cases above the cutoff as high risk and the remainder as low risk, as illustrated in Figure 4. Employing survival analysis, an HR of 2.31 was noted within these two groups. This signifies a notable difference in the risk of mortality between the identified high-risk and low-risk groups. Specifically, individuals in the high-risk category, characterized by a model-derived cutoff of 0.95, exhibited a hazard of mortality that was 2.31 times greater than their low-risk counterparts. This finding underscores the discriminatory power of the model in effectively stratifying patients based on their risk of mortality. Further observations of the survival curves unveiled a distinct separation around days 4 and 5 of monitoring, underscoring the potential of the model to effectively stratify patients based on their risk of mortality in the crucial early days following septic onset. 

## 4. Discussion

Timely identification of sepsis plays a pivotal role in effective clinical management, enabling prompt and precise interventions such as optimal antimicrobial administration and fluid resuscitation. These measures are essential for enhancing the likelihood of survival. Although the onset of sepsis can be acute and result in short-term mortality, it can also be a cause of significant long-term morbidity requiring diagnosis and treatment. Therefore, a multidisciplinary approach to sepsis is necessary to reduce mortality rates through early diagnosis and targeted treatment [1]. The present study delves into the intricate landscape of sepsis through a multifaceted analysis of hematological parameters and immune responses. Our examination of 78 patients and 33 healthy controls provided a comprehensive understanding of the interplay between immune cells, blood cell maturation, and their collective impact on sepsis outcomes. A power calculation using the preliminary dataset yielded levels above 90% for each of the inflammation parameters. This ensured sample sizes were justified in the current study. 

The common challenge in clinical practice for diagnosing sepsis is the time-consuming use of blood cultures to determine the need for immediate empirical antimicrobial therapy. Blood culture results also show positive values in only 30–50% of patient samples with underlying conditions such as diabetes, kidney disease, and advanced age. Therefore, there is a need for biomarkers that can aid in early sepsis detection and monitor its progression to assist in more accurate sepsis management and determine the appropriate treatment to prevent worsening conditions due to sepsis [1]. In a correlated investigation utilizing advanced clinical parameters derived from a hematology analyzer, Ho et al. elucidate crucial prognostic features in cases where cultures yield negative results [26]. It was also noted that the sample sizes utilized were similar, indicating consistency within the hematological parameters used for this study. This emphasizes the potential of advanced hematology analysis not only for its cost-effectiveness but also for its ease of access using a routine CBC test and the rapidity of results compared to traditional culture-based methods. Our study extends the application of these parameters beyond routine reporting, aiming to bolster crucial clinical decision-making, especially in situations where timely interventions are imperative for septic cases.

In our investigation, we assessed the advanced functionalities of automated hematology analyzers, employing innovative gating methods to scrutinize cell populations associated with the activation states of neutrophils and lymphocytes. These parameters offer additional insights into the functional activities of diverse white blood cells in response to sepsis, going beyond the conventional white blood cell count. The EIPs in our study encompass hematological indicators, including neutrophil reactivity intensity (Neut-RI), neutrophil granularity intensity (Neut-GI), antibody-synthesizing lymphocytes (AS-Lymph), and total reactive lymphocytes (RE-Lymph). Neut-RI and Neut-GI reflect neutrophil reactivity, predicting early bacterial infection. Monocyte fractions were evaluated alongside other relevant parameters. While valuable, their inclusion did not lead to a significant enhancement in the AUROC of the current model. Thus, we opted for a simplified model that prioritizes ease of implementation and cost-effectiveness. These parameters are associated with early innate immune responses, with values changing based on the inflammatory process, severity, and infection stage. The body’s natural immune system acts as an initial defense against pathogens, triggering the adaptive immune response, comprising cellular and humoral components. The AS-Lymph parameter quantifies activated B lymphocytes, while RE-Lymph counts all activated lymphocytes, including plasma cells. These parameters were shown to be specific with a high odds ratio in COVID-19 infections [27]. Additionally, when considered alongside absolute counts of immature granulocytes (IGs)—which, in specific infection conditions, revealed distinctions in disease severity [28]—they provide robust evidence to investigate direct alterations in immune response to external insults. 

A robust multiple logistic regression model was devised to integrate specific parameters, showcasing remarkable discriminatory capabilities in effectively discerning septic cases from other groups, as depicted in Figure 3. In this study, the AUROC value for distinguishing septic cases from non-septic cases stood at 0.924. Accompanying this, a negative predictive power of 79.1% and positive predictive power of 85.7% were observed, metrics highly reliant on the prevalence of the disease within the tested population. It is crucial to note that the positive predictive power escalates with increasing prevalence, while the negative predictive power declines. In our hospital setting, particularly within the ICU, the prevalence of sepsis hovers around 30%, potentially influencing the predictive accuracy. However, it is important to highlight that despite this localized prevalence, the overall prevalence in the population remains low. Moreover, besides infections, elevated EIP parameters may also manifest in inflammation related to conditions like diabetes mellitus or coronary artery disease. Hence, the presence of non-communicable diseases in non-septic cases might have contributed to the higher EIPs observed in this study [29,30].

This suggests that, at the initial ICU admission (day 0), patients could benefit from early rule-in or rule-out assessments, preceding the application of confirmatory tests such as SOFA, PCT, APACHE II score, and blood cultures. This observation led to the hypothesis that routine hematological results combined with EIPs could serve as a practical and timely test to aid clinical decisions in sepsis cases. Significantly, these CBC tests impose no additional burden on patients.

Sepsis is a multifaceted condition capable of eliciting various immune responses contingent on its underlying cause. Therefore, relying on a single blood cell parameter may not be the optimal strategy to accurately characterize these patients. A comprehensive examination of the complete CBC profile, particularly focusing on the white blood cell count and EIPs, could provide a more lucid depiction of our immune responses and the activated state of white blood cells in reaction to infection or sepsis. Indeed, by combining the EIPs to assess blood culture-negative cases, this study observed strong associations with clinical outcome prediction, which also identifies the survival probability of patients. Adjusting the cutoff values to enhance the test’s sensitivity for improved outcome stratification is a possibility, but this necessitates a more extensive cohort study and a validation group to evaluate its accuracy. Nonetheless, this study promotes the prompt utilization of these tests by healthcare professionals for the timely detection, diagnosis, and treatment of sepsis. 

This study is not without limitations. The research was conducted in a single center, which might have introduced biases related to specific patient demographics, local practices, or healthcare infrastructure. Multi-center studies would provide a more comprehensive perspective. Although the study examined changes in parameters over time for a small, selected group of septic cases, a more detailed sequential monitoring approach could provide insights into the dynamic nature of immune responses during different phases of sepsis. Nonetheless, this study introduces a novel approach to sepsis management by exploring the combined utility of routine hematological parameters and EIPs. The innovative multiple logistic regression model formulated in this research exhibits formidable discriminatory prowess, offering a timely and practical tool for clinical decision-making in sepsis cases. By delving into the dynamic interplay of immune responses during different phases of sepsis, this study contributes novel insights to the understanding of early sepsis detection and prognostication. The findings not only highlight the potential of routine CBC tests coupled with EIPs but also underscore the need for further exploration and validation of these novel diagnostic approaches in diverse clinical settings.

## 5. Conclusions

In summary, our study provides evidence that the utilization of EIPs may be valuable in diagnosis and predicting patient outcomes, particularly in relation to the activation states of neutrophils and lymphocytes. We observed promising results in stratifying sepsis patients and probability survival after a 30-day monitoring period. However, it is imperative to conduct additional studies with larger sample sizes to validate our findings and establish the clinical relevance of hematological parameters in predicting patient outcomes.

## Figures and Tables

**Table 1:** General data of the patients included in the study group.

Parameter	Sepsis (*n* = 39)	Non-Sepsis (*n* = 39)	Healthy (*n* = 33)	*p*-Value
Age (year)	60.77 ± 14.81	52.97 ± 16.33	33.67 ± 6.14	
Gender				
Male	20 (51.3%)	24 (61.5%)	23 (69.7%)	
Female	19 (48.7%)	15 (38.5%)	10 (30.3%)	
Blood culture				
Positive	5 (12.8%)	0	NA	
Negative	34 (87.2%)	39 (100%)	NA	
Laboratory result				
SOFA	4 (2–12)	0.9 (0–1)	NA	
TWBC (10^3^/µL)	16.84 (5.06–47.60)	7.93 (4.47–20.80)	7.83 (5.55–12.94)	<0.001 ^1^
Neut# (10^3^/µL)	13.40 (3.38–45.35)	1.58 (0.28–3.17)	4.50 (2.60–33.90)	<0.001 ^1^
Neut%	79.10 (44.20–95.20)	67.50 (44.20–93.40)	56.90 (6.40–73.80)	<0.001 ^1^
Mono%	5.89 ± 3.63	7.54 ± 2.76	7.02 ± 1.40	0.0012 ^2^
Lymph# (10^3^/µL)	0.94 (0.15–2.80)	1.58 (0.28–3.17)	2.50 (1.48–4.64)	<0.001 ^1^
Lymph%	8.33 ± 7.06	20.41 ± 11.37	31.86 ± 7.07	<0.001 ^2^
HGB (g/dL)	9.84 ± 3.39	12.17 ± 2.15	14.39 ± 1.37	<0.001 ^2^
PLT# ((10^3^/µL)	224 (13–598)	281 (148–609)	292 (168–424)	0.399 ^1^
RET-He (pg)	30.2 (20.5–37.8)	31.2 (16.0–34.5)	31.7 (23.7–34.6)	0.203 ^1^
RBC-He (pg)	28.7 (23.3–32.0)	29.0 (14.7–33.6)	29.3 (17.3–31.5)	0.737 ^1^
Delta-He (pg)	1.23 ± 3.83	2.35 ± 1.35	2.56 ± 0.90	0.265 ^2^
IG# (10^3^/µL)	0.20 (0.03–3.45)	0.07 (0.01–0.58)	0.08 (0.01–0.21)	<0.001 ^1^
IG%	1.4 (0.3–14.5)	0.8 (0.1–4.7)	0.95 (0.1–1.8)	0.001 ^1^
Neut-RI (FI)	51.1 (40.3–67.8)	46.2 (41.2–61.7)	44.8 (26.9–48.6)	<0.001 ^1^
Neut-GI (SI)	151.6 (138.2–164.4)	147.0 (125.4–159.1)	148.1 (141.0–161.2)	0.001 ^1^
AS-Lymph (10^3^/µL)	0.01 (0.0–0.35)	0.0 (0.0.–0.06)	0.0 (0.0–0.07)	<0.001 ^1^
RE-Lymph (10^3^/µL)	0.10 (0.01–0.50)	0.05 (0.0–0.17)	0.06 (0.0–0.17)	0.026 ^1^

Footnote: Data were presented as mean with standard deviation, median (minimum–maximum), or number with percentage in parenthesis. ^1^ Kruskal–Wallis test, ^2^ one-way ANOVA, *p* < 0.05 was statistically significant. SOFA, sequential organ failure assessment; TWBC, total white blood cell count; Neut#, neutrophil count; Neut%, neutrophils in percentage; Mono%, monocytes in percentage; Lymph#, lymphocyte count; Lymph%, lymphocytes in percentage; HGB, hemoglobin; PLT#, platelet count; RET-He, reticulocyte hemoglobin equivalent; RBC-He, red blood cell hemoglobin; Delta-He, delta hemoglobin; IG# immature granulocyte count; IG%, immature granulocytes in percentage; Neut-RI, neutrophil reactivity intensity; Neut-GI, neutrophil granularity intensity; AS-Lymph, antibody synthesizing lymphocyte; RE-Lymph, reactive lymphocytes.

## Data Availability

The data collected and analyzed during this study are available from the corresponding author upon reasonable request.

## Supplementary Materials

The following supporting information can be downloaded at: https://www.mdpi.com/article/10.3390/diagnostics14090956/s1, Figure S1: Analysis of hematology-extended inflammatory parameters in sepsis and non-sepsis groups. (A) Neut-RI; (B) Neut-GI; (C) AS-Lymph; (D) RE-Lymph.
