# Supplementary material for: Integrating Routine Hematological and Extended Inflammatory Parameters as a Novel Approach for Timely Diagnosis and Prognosis in Sepsis Management
Source: Diagnostics (Basel). 2024 May 2;14(9):956. doi: 10.3390/diagnostics14090956 (PMC11083944; doi:10.3390/diagnostics14090956)
Supplement: Supplementary file 1 [file diagnostics-14-00956-s001.zip › diagnostics-2906526-supplementary.pdf]

diagnostics

MDPI

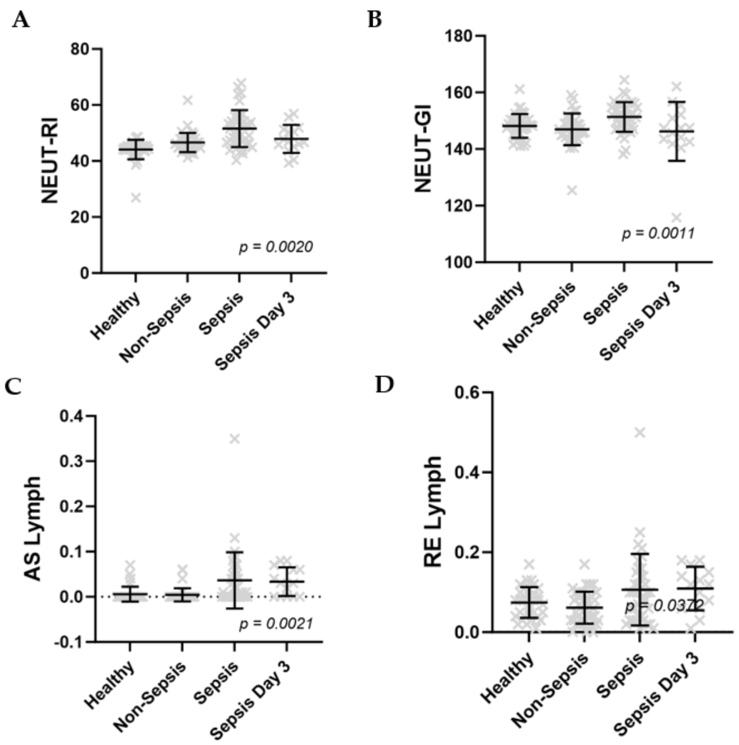

Supplementary Figure S1. Analysis of haematology extended inflammatory parameters in sepsis and non-sepsis groups. (**A**) Neut-RI; (**B**) Neut-GI; (**C**) AS-Lymph (**D**) RE-Lymph.
